# Supplementary material for: Widespread theta synchrony and high-frequency desynchronization underlies enhanced cognition
Source: Nat Commun. 2017 Nov 22;8:1704. doi: 10.1038/s41467-017-01763-2 (PMC5700170; doi:10.1038/s41467-017-01763-2)
Supplement: Supplementary file 1 — Supplementary Information [file 41467_2017_1763_MOESM1_ESM.pdf]

Supplementary Figures

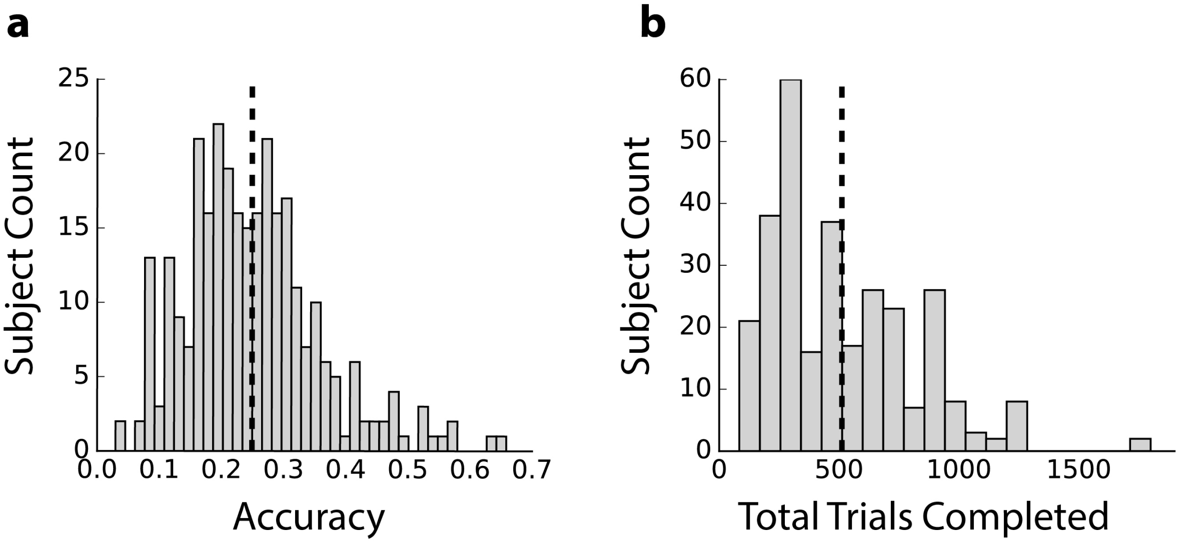

Supplementary Figure 1. Free-recall behavioral results. (a) Distribution of subject accuracy on the verbal delayed free-recall task, mean indicated by vertical line (n=294). **(b)** Distribution of number of total trials (i.e. word presentations) completed by each subject.

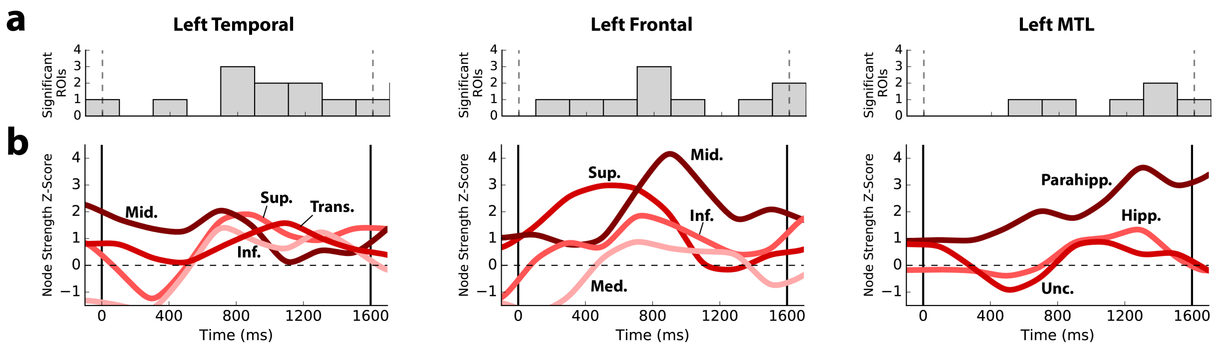

Supplementary Figure 2. Left-hemispheric theta (3-8 Hz) hub timecourses. (Figures organized according to caption of Figure 3 in the main text.) **(a)** Count of significant (P<0.05) node strength for ROIs within three broad regions: left temporal, frontal, and medial temporal lobes. **(b)** Smoothed timecourses of node strength for each ROI.

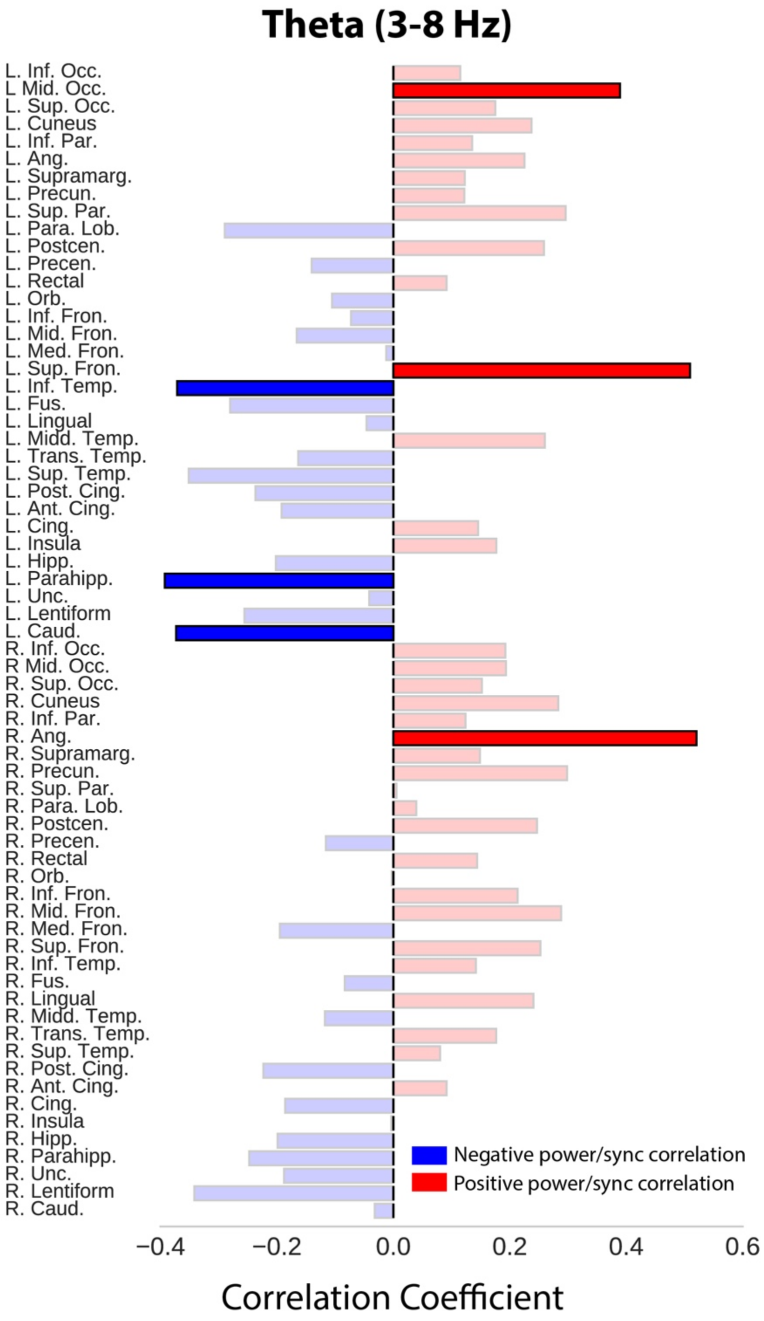

Supplementary Figure 3. Power-synchrony correlation in the theta band. Correlation of the modulation of theta (3-8 Hz) node strength and power across time and frequency for each ROI, during the word encoding interval (0-1600ms). Bar plots show the power-synchrony correlation for each ROI, with blue indicating negative and red indicating positive correlations. Faded bars are not significant after FDR correction for multiple comparisons ($\alpha = 0.05$).

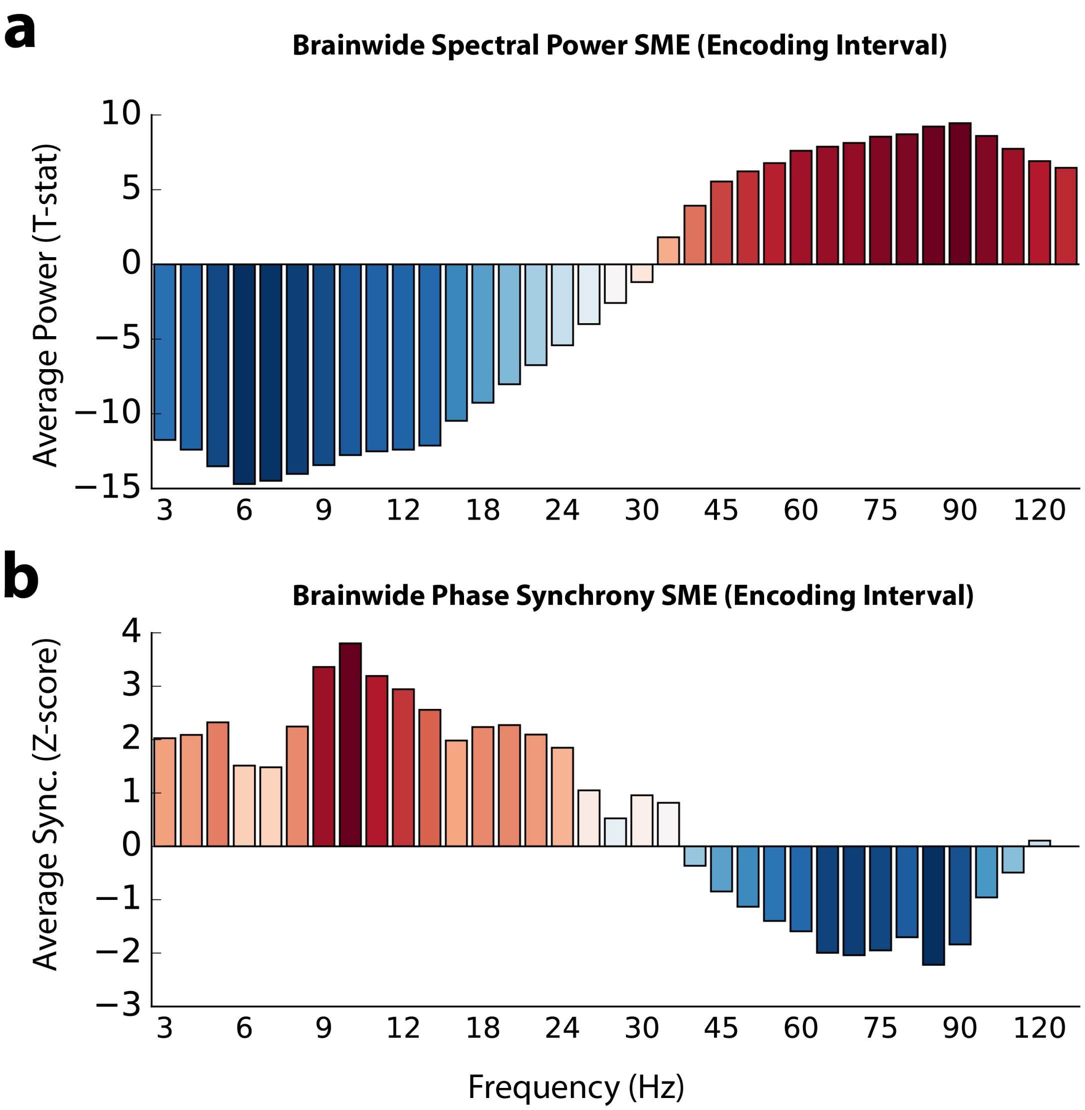

Supplementary Figure 4. Memory-associated brainwide spectral power and phase synchrony in the encoding interval (0-1600 ms). (a) Average brainwide t-statistic reflecting the relative change in spectral power across all electrodes and all subjects in remembered vs. not-remembered conditions (see Fig. S2 and methods for details). **(b)** Average brainwide z-score reflecting the relative change in phase synchrony across all electrode pairs and all subjects in remembered vs. not-remembered conditions.

Supplementary Tables

Region Name	Abbreviation	Z-score
**Core Memory Network (in-network), corrected *P* < 0.01**		
R. Extra-Nuclear	R. Extra-Nuc	N/A
R. Parahippocampal Gyrus	R. Parahipp.	4.11
R. Subgyral	R. Subgyral	N/A
R. Lingual Gyrus	R. Lingual	6.71
R. Fusiform Gyrus	R. Fus.	6.77
R. Middle Frontal Gyrus	R. Mid. Fron.	4.86
R. Inferior Frontal Gyrus	R. Inf. Fron.	5.66
R. Precentral Gyrus	R. Precen.	5.32
R. Superior Parietal Lobule	R. Sup. Par.	3.65
R. Cuneus	R. Cuneus	6.15
R. Superior Occipital Gyrus	R. Sup. Occ.	4.25
R. Middle Occipital Gyrus	R. Mid. Occ.	11.86
R. Inferior Occipital Gyrus	R. Inf. Occ.	11.05
L. Extra-Nuclear	L. Extra-Nuc.	N/A
L. Uncus	L. Unc.	5.07
L. Parahippocampal Gyrus	L. Parahipp.	8.53
L. Hippocampus	L. Hipp.	5.30
L. Cingulate	L. Cing.	4.99
L. Subgyral	L. Subgyral	N/A
L. Superior Temporal Gyrus	L. Sup. Temp.	3.97
L. Middle Temporal Gyrus	L. Mid. Temp.	9.18
L. Lingual Gyrus	L. Lingual	5.46
L. Fusiform Gyrus	L. Fus.	12.21
L. Inferior Temporal Gyrus	L. Inf. Temp.	9.21
L. Superior Temporal Gyrus	L. Sup. Temp.	7.75
L. Medial Frontal Gyrus	L. Med. Fron.	4.40
L. Middle Frontal Gyrus	L. Mid. Fron.	14.31
L. Inferior Frontal Gyrus	L. Inf. Fron.	14.12
L. Orbital Gyrus	L. Orb.	5.04
L. Precentral Gyrus	L. Precen.	4.72
L. Postcentral Gyrus	L. Postcen.	6.13
L. Superior Parietal Lobule	L. Sup. Par.	6.02
L. Supramarginal Gyrus	L. Supramarg.	5.48
L. Inferior Parietal Lobule	L. Inf. Par.	7.69
L. Cuneus	L. Cuneus	6.77
L. Middle Occipital Gyrus	L. Mid. Occ.	12.20
L. Inferior Occipital Gyrus	L. Inf. Occ.	5.67
**Non-Memory Network (out-of-network), corrected *P* > 0.01**		
R. Thalamus	R. Thal.	2.29
R. Caudate	R. Caud.	1.81
R. Lentiform Nucleus	R. Lentiform	0.46

R. Uncus	R. Unc.	-0.37
R. Subcallosal Gyrus	R. Subcall.	0.49
R. Hippocampus	R. Hipp.	1.01
R. Insula	R. Insula	-0.29
R. Cingulate	R. Cing.	1.65
R. Anterior Cingulate	R. Ant. Cing.	0.12
R. Posterior Cingulate	R. Post. Cing.	0.13
R. Superior Temporal Gyrus	R. Sup. Temp.	-2.26
R. Transverse Temporal Gyrus	R. Trans. Temp.	-0.09
R. Middle Temporal Gyrus	R. Mid. Temp.	1.78
R. Inferior Temporal Gyrus	R. Inf. Temp.	0.80
R. Superior Frontal Gyrus	R. Sup. Fron.	0.80
R. Medial Frontal Gyrus	R. Med. Fron.	-1.39
R. Orbital Gyrus	R. Orb.	0.93
R. Rectal Gyrus	R. Rectal	0.94
R. Postcentral Gyrus	R. Postcen.	1.67
R. Paracentral Lobule	R. Para. Lob.	-0.46
R. Precuneus	R. Precun.	0.86
R. Supramarginal Gyrus	R. Supramarg.	-2.30
R. Angular Gyrus	R. Ang.	-1.52
R. Inferior Parietal Lobule	R. Inf. Par.	-0.69
L. Thalamus	L. Thal.	1.64
L. Caudate	L. Caud.	1.35
L. Lentiform Nucleus	L. Lentiform	-0.19
L. Subcallosal Gyrus	L. Subcall.	0.96
L. Insula	L. Insula	1.78
L. Anterior Cingulate	L. Ant. Cing.	-1.29
L. Posterior Cingulate	L. Post. Cing.	2.50
L. Transverse Temporal Gyrus	L. Trans. Temp.	2.23
L. Rectal Gyrus	L. Rectal	2.01
L. Paracentral Lobule	L. Para. Lob.	-1.07
L. Precuneus	L. Precun.	0.17
L. Angular Gyrus	L. Ang.	-1.15
L. Superior Occipital Gyrus	L. Sup. Occ.	2.07

Supplementary Table 1. List of ROIs included in the core memory network. List of the 74 ROIs (37 per hemisphere) used in this study, with abbreviations. Z-scores reflect a comparison of the spectral power between remembered and not-remembered trials, averaged across subjects and electrodes. ROIs in the core memory network had significant power differences between the two conditions ($P < 0.01$, Benjamini-Hochberg corrected for multiple comparisons; see Methods for details). Four subcortical ROIs indicated with N/A were excluded from this analysis due to limited data (less than 5 subjects with electrodes placed in the ROI).
